# Purification and Characterization of Cathepsin B from the Muscle of Horse Mackerel *Trachurus japonicus*

**DOI:** 10.3390/md13116550

**Published:** 2015-10-28

**Authors:** Asami Yoshida, Megumi Ohta, Koichi Kuwahara, Min-Jie Cao, Kenji Hara, Kiyoshi Osatomi

**Affiliations:** 1 Graduate School of Fisheries and Environmental Sciences, Nagasaki University, Nagasaki 852-8521, Japan; E-Mails: megumi-ohta@sea.sannet.ne.jp (M.O.); hara-k@knwu.ac.jp (K.H.); osatomi@nagasaki-u.ac.jp (K.O.); 2 Food Science and Technology Section, Nagasaki Prefectural Institute of Fisheries, Nagasaki 851-2213, Japan; E-Mail: kuwahara012217@pref.nagasaki.lg.jp; 3 College of Food and Biological Engineering, Jimei University, Xiamen 361021, China; E-Mail: mjcao@jmu.edu.cn; 4 Faculty of Food Nutrition, Kyushu Nutrition Welfare University, Kitakyusyu, Fukuoka 803-8511, Japan

**Keywords:** cathepsin B, endogenous protease, horse mackerel, food processing

## Abstract

An endogenous protease in fish muscle, cathepsin B, was partially purified and characterized from horse mackerel meat. On SDS-PAGE of the purified enzyme under reducing conditions, main protein bands were detected at 28 and 6 kDa and their respective N-terminal sequences showed high homology to heavy and light chains of cathepsin B from other species. This suggested that horse mackerel cathepsin B formed two-chain forms, similar to mammalian cathepsin Bs. Optimum pH and temperature of the enzyme were 5.0 and 50 °C, respectively. A partial cDNA encoding the amino acid sequence of 215 residues for horse mackerel cathepsin B was obtained by RT-PCR and cloned. The deduced amino acid sequence contains a part of light and heavy chains of cathepsin B. The active sites and an *N*-glycosylation site were conserved across species. We also confirmed that the *modori* phenomenon was avoided by CA-074, a specific inhibitor for cathepsin B. Therefore, our results suggest that natural cysteine protease inhibitor(s), such as oryzacystatin derived from rice, can apply to thermal-gel processing of horse mackerel to avoid the *modori* phenomenon. Meanwhile, this endogenous protease may be used for food processing, such as weaning meal and food for the elderly.

## 1. Introduction

Lysosomal cysteine proteases, cathepsins B, H, L, and so on, play an important role in intracellular protein turnover. Cathepsin B (EC3.4.22.1) is a major lysosomal cysteine protease and has distinctive characteristics which act not only as an endopeptidase, but also as a carboxyl dipeptidyl peptidase [1]. Therefore, the enzyme is an essential protease for intracellular protein degradation in lysosome.

In marine food science, cathepsin B had been studied as a cause of the fish muscle softening. Muscle softening occurs much faster in fish than in livestock, leading to reduction in commercial value [2,3]. It is generally considered that a major cause of post-mortem muscle softening in fish is proteolysis of muscle structural proteins by endogenous proteases such as lysosomal cysteine proteases. Some researchers reported the relationship between lysosomal cysteine proteases and post-mortem tenderization in fish muscle [4,5,6]. In our previous study, we suggested that α-actinin was degraded by cysteine proteases in red sea bream muscle during storage [4]. Cathepsins B and L of chum salmon hydrolyzed muscle structural proteins with respect to muscle softening [5]. To clarify the mechanism of fish muscle softening, cathepsin B have been purified and characterized from skeretal muscle of common carp [7], silver carp [8], tilapia [9], common mackerel [10], and chum salmon [11].

While cathepsin B is also considered to participate in the gel disintegration during the processing of surimi-based products. Surimi-based products are a seafood product and very popular in Asian countries. It is made from fish muscle proteins, and fish meat is minced with salt to solubilize in the monomer of myofibrillar proteins, such as myosin and actin. Then, solubilized myofibrillar proteins form the network structure by heating and, as a result, the surimi gel is made. However, during heating, formed surimi gel can be destroyed by endogenous proteases in fish muscle; subsequently, quality loss of the gel may occur. It is a general concept that endogenous proteases cause thermal gel-weakening, which is called the *modori* phenomenon. It is one of the urgent problems in the fisheries industry. Consequently, there are some reports about the relationship between endogenous proteases and the *modori* phenomenon. Among the numerous proteinases present in fish muscle, cysteine proteases have the most serious effects on texture of thermal gel because of their thermo-stability and endopeptidase activity [12]. An *et al.* [13] reported that cathepsin L causes degradation of myofibrillar proteins in surimi of Pacific whiting. The common mackerel gel was deteriorated by cysteine proteases such as cathepsin B or L [14]. In addition, myofibril-bound serine proteinase (MBSP) was firstly found in common carp muscle [15] as an endogenous protease responsible for the *modori* phenomenon. MBSPs were also found in the skeletal muscle of lizard fish [16], white croaker [17], yellow croaker [18], silver carp [19], and crucian carp [20].

Horse mackerel, *Trachurus japonicas*, is one of the most important species on seafood processing in Japan, and in particular this species is widely utilized as ingredient for surimi-based products. However, in the horse mackerel, the characteristics of the endogenous proteases and their involvement in *modori* phenomenon are still not clear. In our preliminary study, cathepsin B was the most active cysteine protease in horse makerel muscle at 50 °C, suggesting that the enzyme was likely to participate in thermal gel disintegration. Therefore, the present paper attempted purification, characterization, and molecular cloning of cathepsin B in horse makerel muscle. Moreover, we also investigated if the enzyme was responsible for the *modori* phenomenon. Ultimately the purpose in this study is to contribute to the quality improvement of surimi-based products using natural protease inhibitors and the possible application of endogenous proteases to seafood processing.

## 2. Results and Discussion

### 2.1. Purification of Cathepsin B from Horse Mackerel Meat

**Figure 1:**
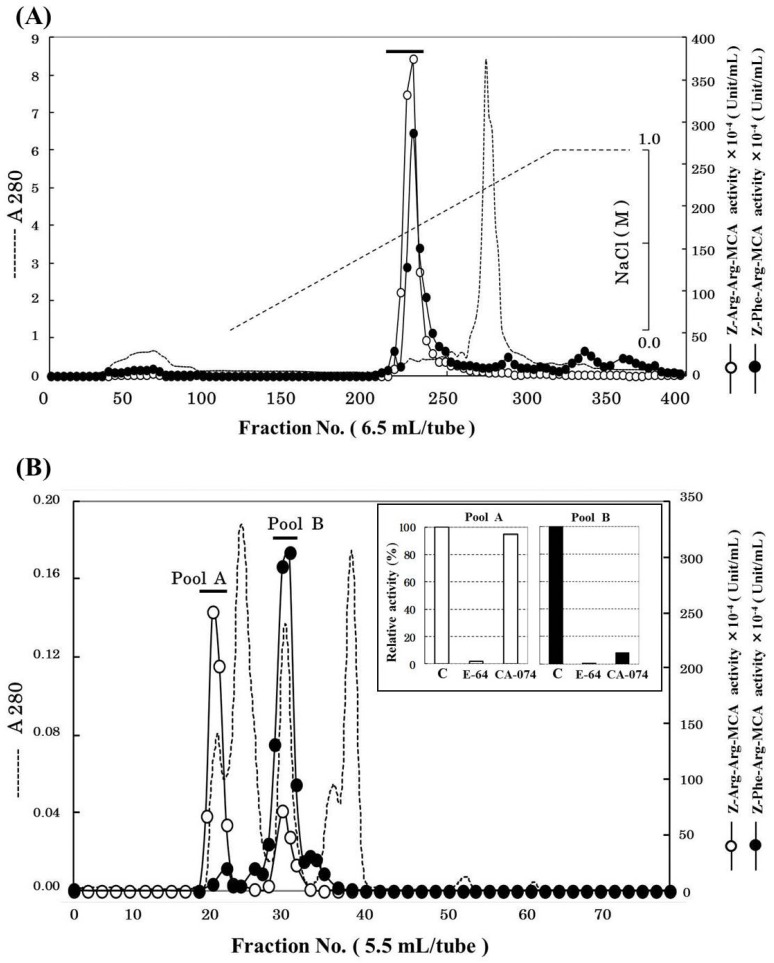
Chromatographic patterns for purification of cathepsin B from horse mackerel meat. (**A**) SP-Sepharose cation-exchange chromatography. The crude enzyme (6732 mg protein) was applied to a column (2.64 × 100 cm), equilibrated with 50 mM acetate buffer (pH 4.5) containing 2 mM 2-mercaptoethanol, and eluted with a linear gradient of NaCl at the concentration of 0 to 1 M in the same buffer; (**B**) Superdex 75 gel filtration chromatography and the effect of CA-074 on the activities of pool A and B from Superdex 75. The enzyme solution (39 mg protein) was applied to a column (320 mL), equilibrated with 50 mM acetate buffer (pH 4.5), containing 2 mM 2-mercaptoethanol and 0.15 M NaCl.

Cathepsin B was partially purified 3132-fold from 1.2 kg of horse mackerel meat with a yield of 1.7% (Table 1) using ammonium sulfate fractionation, cation-exchange chromatography, and gel filtration. The chromatographic profile on SP-Sepharose column is shown in Figure 1A. The active peak was eluted with a linear gradient of 0–0.6 M NaCl and could be separated from most of contaminating proteins. The active fractions were pooled and concentrated by ultrafiltration using YM-10 membrane. And then, the concentrated enzyme was applied to Superdex 75 gel filtration column. As shown in Figure 1B, Z-Arg-Arg-MCA hydrolyzing peak and Z-Phe-Arg-MCA hydrolyzing peaks were separately eluted on the gel filtration column, and two peaks were pooled as “pool A” and “pool B”, respectively. To determine which of the pools was suitable as cathepsin B fraction, the effects of cathepsin B specific inhibitors to both pools were investigated (Figure 1B). The activities of both pools were inhibited by E-64, cysteine protease inhibitor. On the other hands, CA-074, cathepsin B specific inhibitor, only suppressed the activity of pool B but not that of pool A. Hence, we decided that pool B was the cathepsin B fraction, and it was used for further purification. We tried to purify the protease from pool A separately; however, we could not identify it because of the decrease its activity due to freeze and thaw cycles.

**Table 1:** Summary of purification of cathepsin B from horse mackerel muscle.

Purification Step	Total Protein (mg)	Total Activity (×10^−4^ U) *	Specific Activity (×10^−4^ U/mg)	Purity (fold)	Yield (%)
Crude enzyme	61,827.35	88,272.26	1.43	1	100.0
50%–70% (NH_4_)_2_SO_4_	6732.00	26,081.32	3.87	3	29.5
SP-Sepharose	39.00	62,231.00	1595.67	1118	70.5
Superdex 75	1.37	2512.24	1832.51	1284	2.8
Mono S	0.34	1520.14	4470.99	3132	1.7

* The activities of the purified cathepsin B were measured using Z-Phe-Arg-MCA at 50 °C for 8 min in 50 mM acetate buffer (pH 5.0).

**Figure 2:**
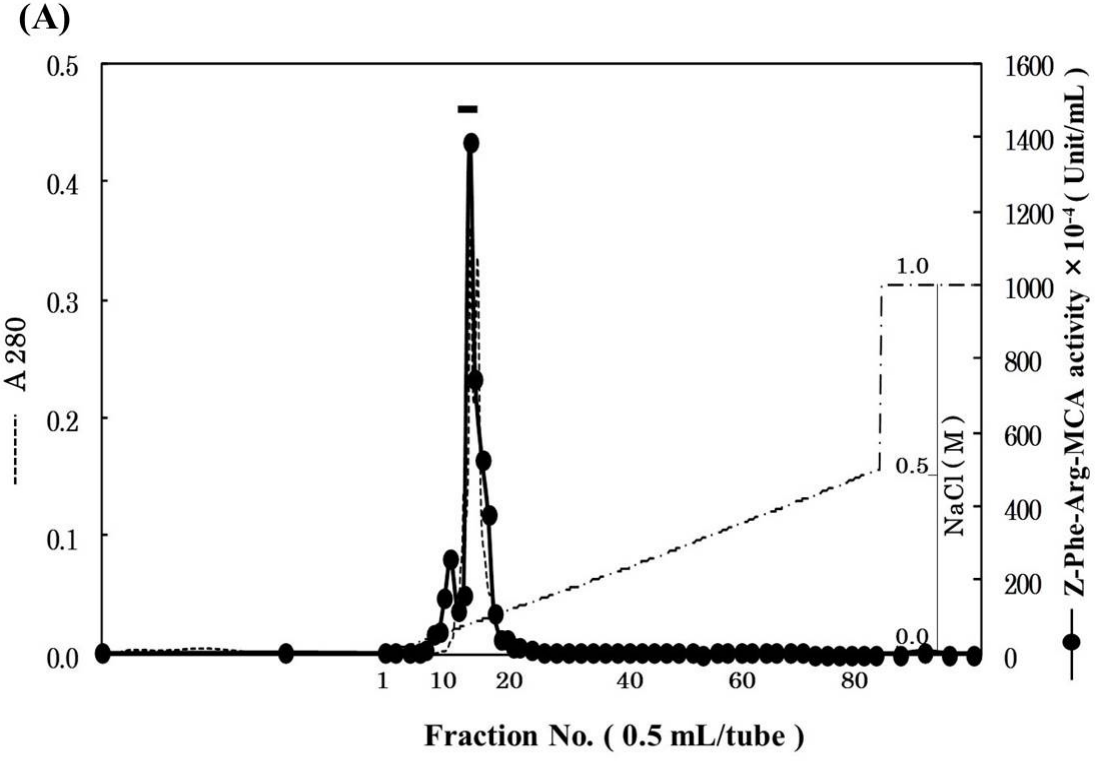

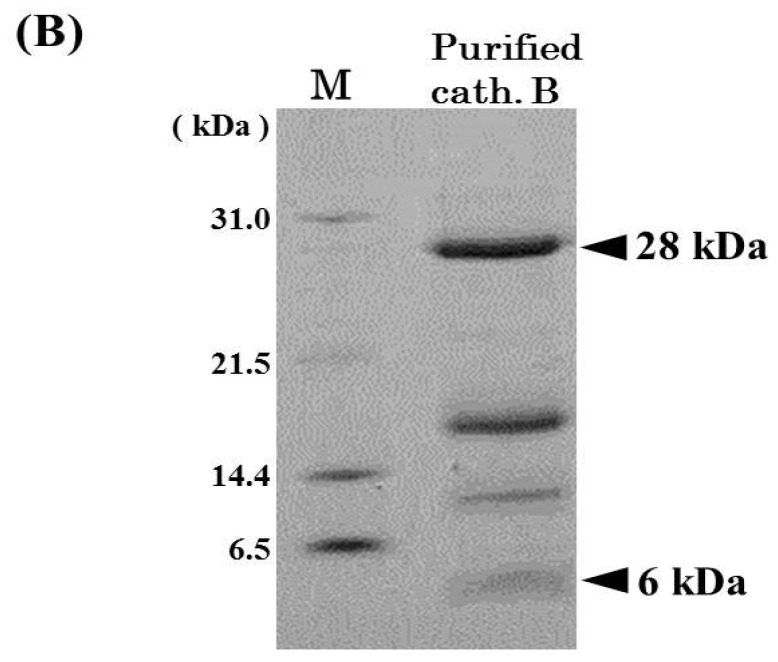
Mono S cation-exchange chromatography (**A**) and SDS-PAGE (**B**) patterns of cathepsin B from horse mackerel muscle. (**A**) The enzyme solution (1.37 mg protein) was applied to a column (1.7 mL), equilibrated with 50 mM acetate buffer (pH 4.5) containing 2 mM 2-mercaptoethanol, and eluted with a linear gradient of NaCl at the concentration of 0 to 0.5 M in the same buffer, and then eluted with a step wise of NaCl at the concentration of 0.5 to 1.0 M in the same buffer; (**B**) The purified cathepsin B fraction (18 µg protein) was applied to SDS-PAGE under reducing conditions on a 10% gel and stained by CBB. Arrowheads indicate protein bands of cathepsin B polypeptides. M, molecular weight protein standards were loaded. Protein standards was composed of carbonic anhydrase (M.W. 31,000), trypsin inhibitor (M.W. 21,500), lysozyme (M.W. 14,400), and aprotinin (M.W. 6500).

**Figure 3:**
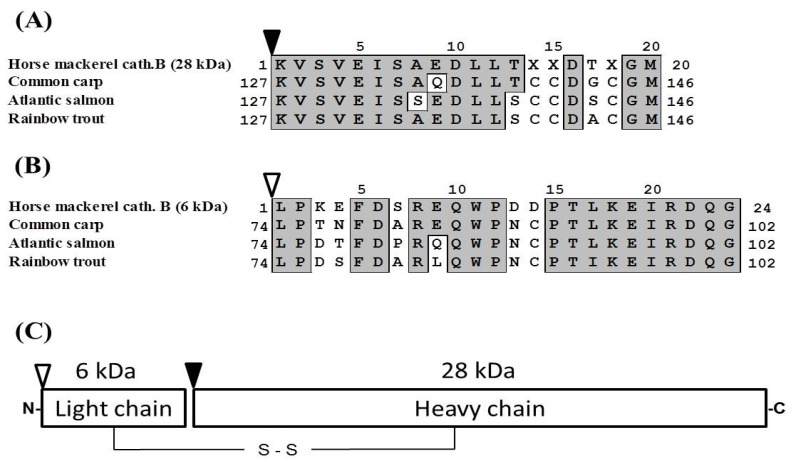
Alignment of N-terminal amino acid sequences of heavy (**A**) and light (**B**) chains of cathepsin B. (**C**) Schematic representation of predicted structure of horse mackerel cathepsin B. Black arrowhead indicates N-terminal of heavy chain of cathepsin B. White arrowhead indicates N-terminal of light chain of cathepsin B. Numbers indicate positions of each of the amino acid sequences. Identical residues with horse mackerel cathepsin B are boxed and shaded in gray. Cathepsin B from common carp (GenBank accession number: AB215097.1), Atlantic salmon (BT058506.1), and rainbow trout (NM001124304.1).

Pool B was applied to Mono S cation-exchange column, and the active peak was obtained (Figure 2A). Since the active peak corresponded to a protein peak, the peak was analyzed by SDS-PAGE to confirm its purity and subunit structure (Figure 2B). On SDS-PAGE under reducing conditions, four protein bands were detected and their N-terminal amino acid sequences were tried to be determined by Edman degradation. We could determine the sequences of 28 kDa and 6 kDa bands for 20 and 24 residues, respectively. While, because of N-terminal blocking or other reasons, the sequences of other two bands could not be determined, and these two bands were assumed to be contaminating proteins. From the results of a homology search, the sequences of 28 and 6 kDa subunits revealed high homology to a heavy chain and a light chain of cathepsin B from other fish (common carp, Atlantic salmon, rainbow trout), respectively (Figure 3A,B). Therefore, it was suggested that horse mackerel cathepsin B was composed of a light chain and a heavy chain with a disulfide bond as shown in Figure 3C. Carp cathepsin B was also reported to be a two-chain form [7]. The processing mechanism of cathepsin B in mammals has been clarified [21] and the single-chain form was converted to two-chain form by cysteine proteinases in lysosome [22]. In this study, horse mackerel cathepsin B was found to be two-chain form in muscle as with other species.

### 2.2. Effects of pH and Temperature on Cathepsin B Activity

**Figure 4:**
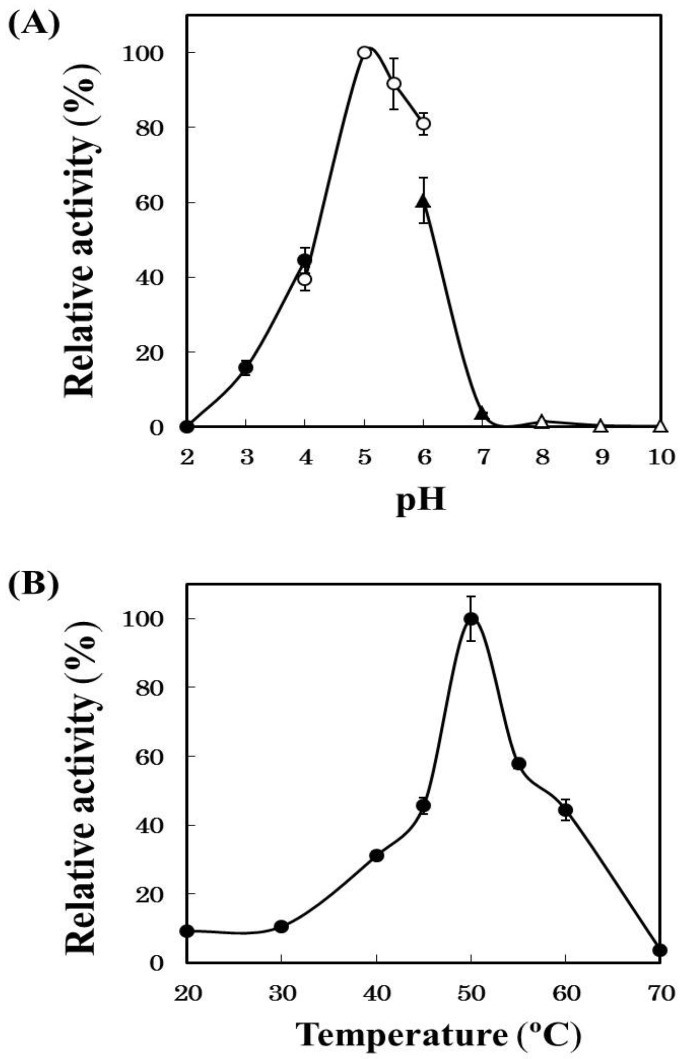
Effects of pH (**A**) and temperature (**B**) on the activity of the purified cathepsin B. (**A**) The activities of the purified cathepsin B were measured with Z-Phe-Arg-MCA at 50 °C using different buffers (pH 1.5~5.0, 0.2 M HCl-CH_3_COOH buffer; pH 5.0~6.0, 0.2 M CH_3_COOH-CH_3_COONa buffer; pH 6.0~8.0, 0.2 M KH_2_PO_4_-Na_2_HPO_4_ buffer; pH 8.0~10.0, 0.2 M boric acid + KCl-Na_2_CO_3_ buffer); and (**B**) the activities of the purified cathepsin B were measured at various temperatures at pH 5.0 using Z-Phe-Arg-MCA.

Horse mackerel cathepsin B was examined the effects of pH and temperature using Z-Phe-Arg-MCA as a substrate (Figure 4). Optimum pH of the enzyme was 5.0 and the activity remained about 60% at pH 6.0 which was the similar pH as the minced meat of horse mackerel. Optimum temperature of the enzyme was 50 °C and the activity remained at least 40% at 60 °C, namely a temperature induced *modori* phenomenon on horse mackerel meat. These results supported our hypothesis that cathepsin B was involved in the deterioration of surimi-based products of the horse mackerel meat. While, in common mackerel [14] and blue scad [23], it has been reported that cathepsin L mainly participate in gel-weakening. Thus, the types of endogenous protease responsible for *modori* phenomenon vary depending on fish species.

### 2.3. Effects of Protease Inhibitors on Cathepsin B Activity

Cathepsin B activity was almost completely inhibited by E-64, CA-074, and chymostatin (Table 2). These inhibitors are cysteine protease inhibitors, and especially CA-074 is a cathepsin B specific inhibitor. Other protease inhibitors we tested did not give any significant effects to the enzyme activity compared with those.

**Table 2:** Effects of protease inhibitors on the activity of purified cathepsin B.

Reagents	Concentration (mM)	Relative Activity (%) *
Control		100
E-64	0.01	1
CA-074	0.01	3
Chymostatin	0.10	4
Pefabloc SC	1.00	89
Pepstatin A	0.01	103
EDTA	1.00	122
2-Mercaptoethanol	2.00	175

* The activities of the purified cathepsin B were measured using Z-Phe-Arg-MCA at 50 °C for 8 min in 50 mM acetate buffer (pH 5.0) with various inhibitors.

### 2.4. Substrate Specificity of Purified Cathepsin B

The enzyme mainly hydrolyzed Z-Phe-Arg-MCA, cathepsin B specific substrate (Table 3). While, another cathepsin B specific substrate, Z-Arg-Arg-MCA, was hydrolyzed by the enzyme for 17% compared with 100% of Z-Phe-Arg-MCA. These results showed the typical characteristic of cathepsin B as with common carp [7,24] and chum salmon [11].

**Table 3:** Substrate specificity of purified cathepsin B.

Substrates	Relative Activity (%) *
Z-Phe-Arg-MCA	100
Z-Arg-Arg-MCA	17
Arg-MCA	0
Boc-Phe-Ser-Arg-MCA	29
Boc-Val-Pro-Arg-MCA	8
Suc-Leu-Leu-Val-Tyr-MCA	6

* The activities of the purified cathepsin B were measured at 50 °C for 8 min in 50 mM acetate buffer (pH 5.0) using various MCA substrates.

### 2.5. Molecular Cloning of Cathepsin B from Horse Mackerel

**Figure 5:**
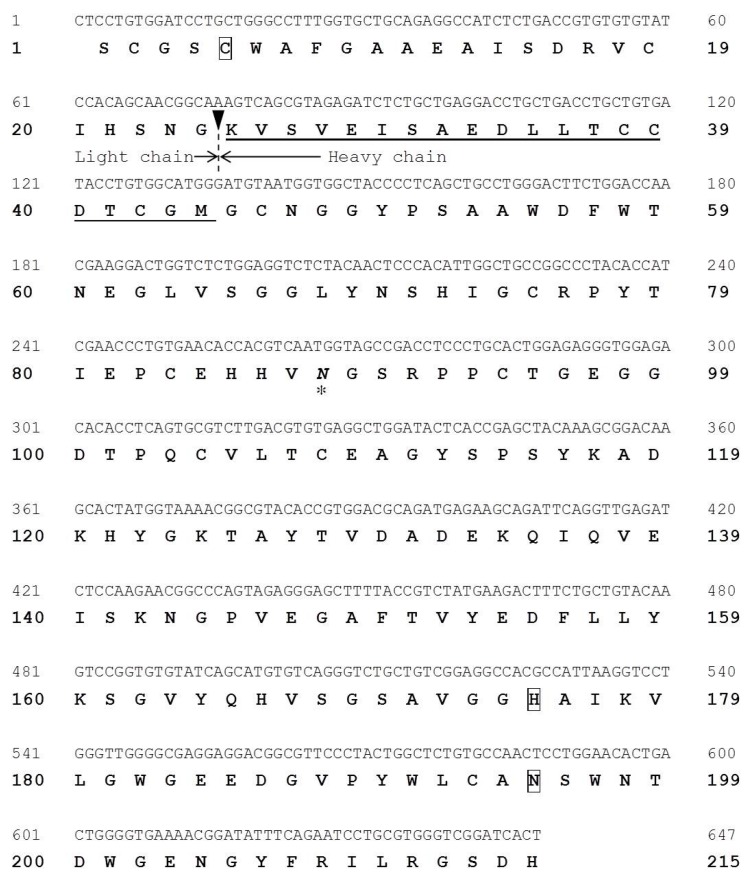
Nucleotide and deduced amino acid sequences of horse mackerel cathepsin B cDNA (GenBank accession number: LC093263). The N-terminal amino acid sequence of purified cathepsin B is underlined. The active site residues of cathepsin B are boxed. Asterisk indicates predicted *N*-glycosylation site. The cleavage site between light and heavy chains is indicated by an arrowhead.

We cloned a partial cDNA corresponding to cathepsin B from the horse mackerel muscle by RT-PCR. The nucleotide and deduced amino acid sequences of cathepsin B cDNA (647 nucleotides) are shown in Figure 5. The sequence consisted of a 647 bp nucleotide sequence encoding 215 amino acids. The cleavage site between the light and heavy chains of cathepsin B was confirmed by N-terminal amino acid sequence of the purified cathepsin B from horse makerel muscle (black arrowhead in Figure 5). This result shows slightly difference in other species, suggesting that there may be a variation in the enzyme responsible for cleaving between light and heavy chains.

**Figure 6:**
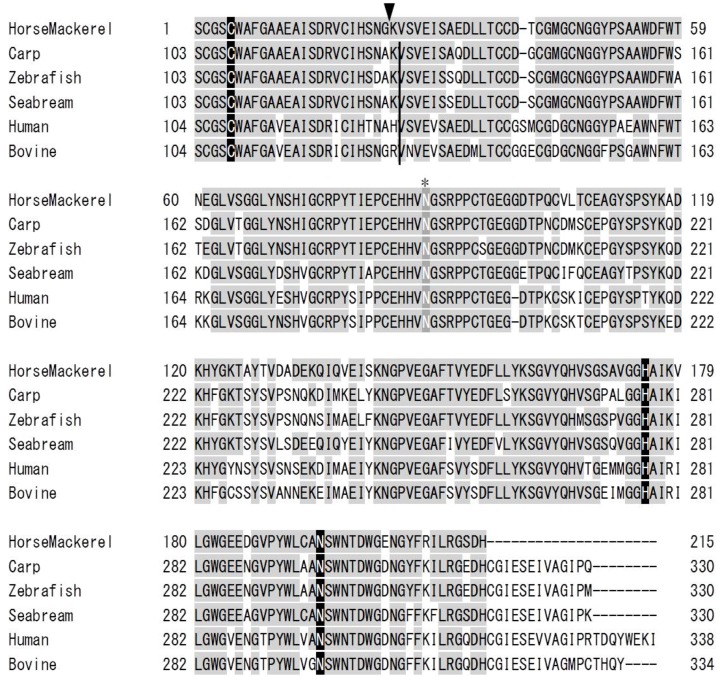
Amino acid sequence alignment of horse mackerel and other cathepsin Bs. Identical residues with horse mackerel cathepsin B are shaded in gray. The active site residues of cathepsin B (Cys, His, Asn) are shaded in black with white letters. Asterisks indicate predicted *N*-glycosylation site. The cleavage site between light and heavy chains in other species is indicated by a black vertical line. Black arrowhead indicates N-terminal of heavy chain of cathepsin B from horse mackerel. Cathepsin B from common carp (GenBank accession number: AB215097.1), zebrafish (NP_998501.1), sea bream (AHZ34284.1), human (AAH95408.1), and bovine (NP_776456.1).

A sequence homology search of the deduced amino acid sequence of horse mackerel cathepsin B was performed on the NCBI Protein database with BLASTP and the multiple sequence alignment of the enzyme and cathepsin Bs from other species was shown in Figure 6. The deduced amino acid sequence was compared with cathepsin B from common carp (GenBank accession no.: AB215097.1), zebrafish (NP_998501.1), sea bream (AHZ34284.1), human (AAH95408.1), and bovine (NP_776456.1), which shared 84.2%, 83.3%, 86.0%, 75.8%, and 76.7% identities, respectively. The active sites and an *N*-glycosylation site of horse mackerel cathepsin B were conserved in cathepsin Bs from other species.

### 2.6. Effects of Cathepsin B Specific Inhibitors on Modori Gel

**Figure 7:**
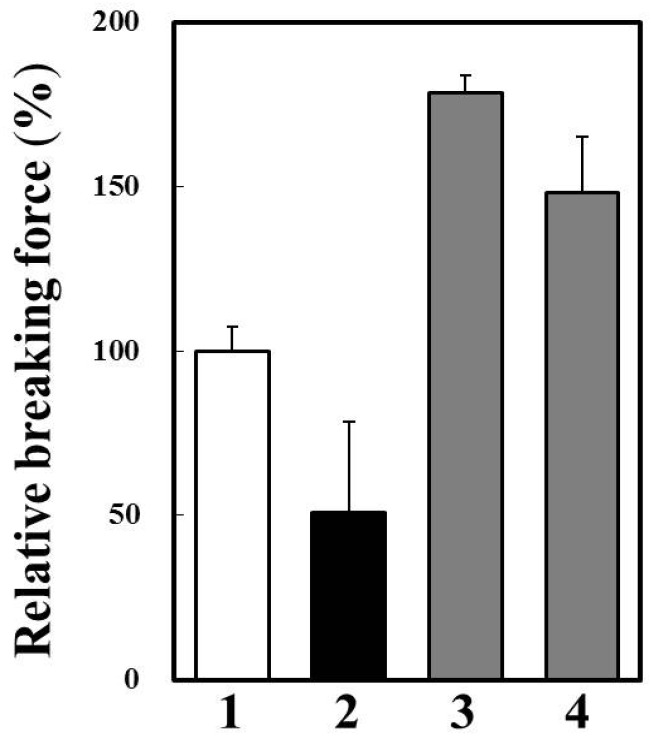
Effects of cysteine protease inhibitors on *modori* gel prepared from horse mackerel meat. **1**, one-step heated gel at 90 °C for 20 min; **2**, *modori* gel without protease inhibitor; **3**, *modori* gel with 0.01 mM E-64; and **4**, *modori* gel with 0.01 mM CA-074.

We prepared *modori* gel of horse mackerel meat with or without cathepsin B inhibitors (Figure 7). Breaking force of *modori* gel was significantly lower than that of control gel. A cysteine protease inhibitor, E-64, and a cathepsin B specific inhibitor, CA-074, dramatically improved breaking force of *modori* gels. Therefore, it was suggested that cathepsin B caused the *modori* phenomenon in horse mackerel thermal gel. Since we confirmed that cathepsin B was the target endogenous protease to avoid disintegration of horse mackerel gel, it is possible to use oryzacystatin as a food additive upon thermal gel processing. Oryzacystatin is a natural cysteine protease inhibitor from rice [25] and is studied about its utilization as a food additive on thermal gel of walleye pollock [26]. The effect of oryzacystatin on *modori* gel of horse mackerel should be clarified for the further study. On the other hand, the endogenous proteases which have high activity and thermal stability, can be used for soft food processing without chemical additive. In this case, the autolytic activity of cathepsin B in horse mackerel muscle has a possibility for application to make soft foods, such as weaning meal and food for the elderly.

## 3. Experimental Section

### 3.1. Fish

The minced meat of horse mackerel, *Trachurus japonicas*, was purchased from Nagasaki kamaboko marine products processing industry cooperative (Nagasaki, Japan). The minced muscle was used for protein purification and RNA extraction of cathepsin B.

### 3.2. Chemicals

SP-Sepharose, Superdex 75, and Mono S were obtained from GE healthcare (Uppsala, Sweden). Fluorogenic peptide substrates, E-64, and Pepstatin A were purchased from Peptide Institute (Osaka, Japan). Pefabloc SC was purchased from Merck KGaA (Darmstadt, Germany). Protein standards for SDS-PAGE were from Bio-rad (Hercules, CA, USA). All other chemicals used were of analytical grades.

### 3.3. Assay of Protease Activity

Cathepsins B activity was assayed using Z-Phe-Arg-MCA and Z-Arg-Arg-MCA as substrates according to the method of Tan [27]. The reaction mixture consisted of 250 μL of 0.1% Brij-35, 200 μL of 0.4 M acetate buffer (pH 5.5) containing 5 mM EDTA, 100 μL of 20 mM cysteine, and 250 μL of the enzyme solution making a total volume of 0.8 mL. The mixture was preincubated at 37 °C for l min, and then incubated again for 10 min under the same condition after the addition of 200 μL of 25 μM substrate. The reaction was terminated by adding 1.5 mL of 0.1 M sodium acetate buffer (pH 4.3) containing 0.1 M sodium monochloroacetate. The fluorescence of 7-amino-4-methylcoumarin (AMC) liberated by enzyme hydrolysis was measured in a spectrofluorometer (RF-1500: Shimadzu Co., Kyoto, Japan) at an excitation wavelength of 380 nm and an emission wavelength of 450 nm. One unit of the enzyme activity was defined as 1 nmol of AMC released per min.

### 3.4. Purification of Cathepsin B

Horse mackerel meat (about 1.2 kg) was homogenized with four volumes of the extraction buffer (50 mM phophate buffer, pH 6.0, containing 2 mM β-mercaptoethanol) and centrifuged at 13,000× *g* for 30 min. The obtained supernatant was as the crude enzyme and was fractionated by ammonium sulfate precipitation (50%–70% saturation). The ammonium sulfate fraction was applied to SP-Sepharose cation-exchange column. Subsequently, the cathepsin B active fraction of SP-Sepharose was purified by Superdex 75 gel filtration column and Mono S cation-exchange column. All purification steps were performed at 4 °C. Cathepsin B was examined for purity using sodium dodecyl sulfate-polyacrylamide gel electrophoresis (SDS-PAGE).

### 3.5. Sodium Dodecyl Sulfate-Polyacrylamide Gel Electrophoresis (SDS-PAGE)

SDS-PAGE was performed according to the method of Laemmli [28] using 10% polyacrylamide gel. After the run, the gel was stained with Coomassie Brilliant Blue R-250 and destained.

### 3.6. Measurement of Protein Concentration

Protein in column eluates was determined by measuring the absorbance at 280 nm. The protein concentration was determined according to the method of Lowry [29] using bovine serum albumin as the standard.

### 3.7. Determination of N-terminal Amino Acid Sequence

The purified enzyme was applied to SDS-PAGE, and transferred to a polyvinylidene difluoride (PVDF) membrane. After brief staining with Coomassie Brilliant Blue R-250, the protein bands were excised, destained, and analyzed by PPSQ-33A protein sequencer (Shimadzu Co., Kyoto, Japan).

### 3.8. Total RNA Isolation and cDNA Cloning by RT-PCR

The fresh ordinary muscle from the horse mackerel was excised and immediately soaked in five volumes of RNAlater (Ambion, Austin, TX, USA) to stabilize the RNA in the tissue. Then, total RNA was isolated from the tissue by ISOGEN (Nippongene, Tokyo, Japan) according to the manufacturer’s protocol.

The first-stranded cDNA was synthesized as a template for RT-PCR. The RNA was denatured and subsequently reverse-transcribed with SuperScript II RT (Invitrogen, Carlsbad, CA, USA) and the oligo-dT primer (CDS-BR: 5′-GGCCACGCGTCGACTAGTAC(T)_16_-3′) containing adapter primer sequence. The primers were removed from the reaction mixture by Microcon-100 (Millipore, Billerica, MA, USA) and finally the first-stranded cDNA pool was obtained. For RT-PCR, two degenerated primers, a sense primer (5′-AGGAGATCAGNGAYCARGG-3′) and an antisense primer (5′-CAATYTCRGAYTCDATNCCAC-3′), were designed based on the N-terminal amino acid sequences of purified cathepsin B protein and the conserved sequence in other cathepsin Bs, respectively. Veriti Thermal Cycler and AmpliTaq Gold (Applied Biosystems, Foster, CA, USA) were used for amplification and the reaction was performed with 1 cycle of 10 min at 95 °C, 35 cycles of 1 min at 94 °C, 1 min at 55 °C, 2 min at 72 °C, and 1 cycle of 7 min at 72 °C. The PCR products separated by agarose gel electrophoresis were extracted from the gel using QIAEX II Gel Extraction kit (QIAGEN, Tokyo, Japan) and cloned into pGEM T-Easy Vector (Promega, Madison, WI, USA). Additionally, sequences of each clone were verified by DNA sequencing.

### 3.9. DNA Sequencing and Analysis

DNA sequencing was carried out by the dideoxy chain termination method using BigDye Terminator v3.1 Cycle Sequencing Kit (Applied Biosystems) with 3130 Genetic Analyzer (Applied Biosystems). Sequence analysis was performed with MacVector software (MacVector, Cary, NC, USA), the BLAST and the domain search programs on DDBJ/GenBank/EMBL databases.

### 3.10. Preparation and Evaluation of Thermal Gel

Minced horse mackerel meat was ground for 3 min by Stephan vacuum cutter UM-5 (Stephan: Hameln, Germany) with 3% sodium chloride. The *surimi* paste was packed into a polyvinylidene chloride tube (diameter: 27 mm) and heated in a water bath at 90 °C for 20 min as a control or at 60 °C for 120 min to induce disintegration of the gel as a *modori* gel. After heating, the gel was immediately cooled into cold water and kept on ice before the measurement of gel strength.

Evaluation of quality of the gels was carried out by measuring their gel strength. The gels were sliced into 15 mm and their breaking force (N) on a Rheoner creep meter RE3305 equipped with a spherical plunger (diameter: 5 mm) (Yamaden: Tokyo, Japan) with a rising rate of the sample table at 6 cm/min. Triplicate measurements were made and the mean value was presented.

## 4. Conclusions

This paper was the first report that cathepsin B was purified and characterized from horse mackerel muscle. The partial cDNA sequence of it was also determined. The characteristics of the enzyme from horse mackerel were similar to other species cathepsin Bs. In addition, the gel strength of *modori* gel was increased by suppression of cathepsin B activity using CA-074, from these results, cathepsin B may cause *modori* phenomenon. Therefore, our results suggest that natural cysteine protease inhibitor(s), such as oryzacystatin derived from rice may apply to *surimi*-based product processing of horse mackerel to improve the quality of thermal gels. On the other hands, the further study will also be needed to utilize endogenous proteases for soft food processing, such as weaning meal and food for the elderly.
